# Efficacy of golimumab in patients with refractory non-infectious panuveitis

**DOI:** 10.1038/s41598-024-52526-1

**Published:** 2024-01-25

**Authors:** Usanee Tungsattayathitthan, Nattaporn Tesavibul, Pitipol Choopong, Chaipat Treeratsakulchai, Yaninsiri Ngathaweesuk, Wilawan Sanphan, Sutasinee Boonsopon

**Affiliations:** 1 Department of Ophthalmology, Faculty of Medicine Siriraj Hospital, Mahidol University, 2 Wanglang Road, Bangkoknoi, Bangkok, 10700 Thailand; 2 Department of Ophthalmology, Phramongkutklao College of Medicine, Bangkok, Thailand

**Keywords:** Medical research, Outcomes research

## Abstract

This study investigated the efficacy of golimumab in the management of refractory non-infectious panuveitis. Nineteen patients (38 eyes; mean age, 31 years) were retrospectively reviewed between June 2016 and June 2022. All patients had bilateral eye involvement and Behçet’s disease was the most common diagnosis (57.9%). Compared to the period before golimumab treatment, the rate of uveitis relapses after golimumab treatment significantly decreased from 1.73 to 0.62 events per person-years (incidence ratio 0.33, 95% confidence interval 0.19–0.57, *P *< 0.001). After golimumab therapy, 12 patients (63.2%) were able to reduce the number or dosage of immunosuppressive drugs, and the median dosage of systemic corticosteroids was reduced from 15.0 to 7.5 mg/d (*P *= 0.013) compared to baseline. The median logMAR visual acuity improved from 0.9 at baseline to 0.6 at the last visit (*P *= 0.006). Golimumab demonstrated efficacy against refractory non-infectious panuveitis in terms of a corticosteroid-sparing effect and reduced the rate of uveitis relapses to approximately one-third.

## Introduction

Non-infectious uveitis (NIU) affects individuals of all ages, and recent evidence indicates that patients with active or refractory NIU are at significant risk of visual impairment, ocular complications, and blindness. Patients with Behçet’s disease and active uveitis are at a 2.5 times higher risk of developing visual impairment and a 2.7 times higher risk of developing blindness, compared to those with inactive uveitis^1^. Moreover, recurrent or persistent inflammation is associated with a risk for ocular complications such as cataracts, glaucomas, and retinal disorders^2^. Therefore, NIU patients with this condition often require chronic treatment using corticosteroids with immunomodulatory therapy (IMT) to effectively achieve the quiescent stage of inflammation while minimising ocular and systemic adverse effects. Conventional clinically used IMTs to treat uveitis include antimetabolites, calcineurin inhibitors, and alkylating agents. If inflammation is unresponsive to these IMTs or a specific diagnosis such as Behçet’s disease was established, biologics including tumour necrosis factor-alpha (TNF-⍺) inhibitors are considered based on expert panel recommendations^3^.

The two anti-TNF-⍺ agents adalimumab and infliximab have been shown in various studies to effectively treat NIU^4^. Golimumab (GLM) stands as another developed anti-TNF-⍺ agent, presenting as a fully human monoclonal antibody. In GLM, the constant regions of both the heavy and light chains share identical amino acid sequences with those observed in infliximab. However, unlike infliximab, where the variable regions of both heavy and light chains are derived from mice, GLM's corresponding variable regions are of human origin. This distinction empowers GLM to reduce the risk of inducing neutralizing antibodies, subsequently minimizing the likelihood of allergic reactions. Moreover, its monthly subcutaneous administrations enable less frequent dosing^5^. The available evidence from a systematic review and meta-analysis suggests that GLM may have potential efficacy in treating NIU; however, six out of eight case series were conducted in patients with anterior uveitis, consisting of juvenile idiopathic arthritis and spondyloarthritis cases^6–12^. To our knowledge, studies reporting the efficacy of GLM in treating panuveitis are limited to a few case reports and case series^13–16^.

Here, we aimed to assess the efficacy of GLM in treating refractory non-infectious panuveitis. This study evaluated the rates of uveitis relapse, changes in visual acuity (VA), corticosteroid dosage adjustments, and IMT use in response to GLM treatment.

## Methods

### Study design and eligibility criteria

We reviewed the medical records of patients with refractory non-infectious panuveitis who were treated with at least 6 monthly subcutaneous injections of GLM between June 2016 and June 2022 at our uveitis clinic, Siriraj Hospital, Mahidol University, Bangkok, Thailand. The inclusion criteria were patients with non-infectious panuveitis who had experienced a relapse of ocular inflammation despite previous treatment with corticosteroids and at least one IMT. Additionally, we included patients with intraocular inflammation not controlled by a prednisolone-equivalent dosage of < 10 mg/d combined with at least one IMT. Patients who received fewer than six injections of GLM were excluded from the study. If patients changed their treatment regimen to other anti-TNF-⍺ agents, we counted their last visit before this change. This study was approved by the Siriraj Institutional Review Board (COA no. Si 686/2022) following the tenets of the Declaration of Helsinki and registered with the Thai Clinical Trials Registry (TCTR 20200515002).

### Data collection

The collected data included demographic information, uveitis diagnosis, clinical eye examination, duration of uveitis before receiving GLM, IMT use before GLM treatment, and corticosteroid and IMT dosages. Uveitis anatomical classification, course of uveitis, anterior chamber cells, and vitreous haze were graded based on the Standardization of Uveitis Nomenclature (SUN) criteria^17^. Best-corrected visual acuity (BCVA), anterior chamber cells, vitreous haze, posterior segment findings, and corticosteroid and IMT dosages were recorded at baseline, month 3, month 6, month 12, and the last visit of GLM therapy. GLM (Simponi^®^, Jansen Biotech Inc., USA) was administered according to the standard regimen by subcutaneous injection of 50 mg every four weeks. The baseline date referred to the day GLM was first prescribed, and the last visit date referred to the last day within one month of GLM discontinuation. Adverse drug events and treatment complications were assessed and recorded.

Relapse of uveitis was defined as the presence of new keratic precipitates, a two-step increase in the level of anterior chamber cells, vitreous haze, an increase from grade 3 + to 4 +, or new-onset retinitis/choroiditis. Uveitis relapse dates were collected throughout the follow-up period, before and during GLM treatment. According to the SUN classification, remission was defined as grade 0 anterior chamber cells for ≥ 3 months after discontinuing all uveitis treatments^17^. We included the absence of active keratic precipitates and active retinitis/choroiditis as additional remission criteria.

BCVA was measured using the Early Treatment Diabetic Retinopathy Study chart and converted to logMAR before analysis; logMAR values of 2.2, 2.3, 2.4, and 2.5 represent a VA of counting fingers, hand motion, light perception, and no light perception, respectively^18^.

### Outcome measures

The main outcome measure was uveitis relapse occurrence, including the overall rates of uveitis relapse before and during GLM treatment and the factors associated with the first uveitis relapse after GLM treatment. Secondary outcomes included changes in BCVA, daily systemic and topical prednisolone dosages, and IMT use between baseline and last visit for GLM therapy.

### Statistical analyses

Descriptive statistics were used to summarize the patient demographics and characteristics. Categorical data are expressed as numbers and percentages, whereas continuous data are presented as mean ± standard deviation (SD) or median (interquartile range; IQR), depending on the data distribution. Changes in logMAR BCVA and daily dosage of systemic and topical prednisolone from baseline to each visit were assessed using Wilcoxon’s signed-rank test.

Uveitis relapse rates before and during GLM treatment were calculated as the number of events per person-year (PY) and compared using the Poisson regression model from generalised estimating equations. The probability of the first uveitis relapse after GLM treatment was analyzed using Kaplan–Meier survival analysis, censoring patients without events at the last known follow-up. The Cox proportional hazards model was used to identify variables independently associated with the first uveitis relapse. All statistical analyzes were performed using IBM SPSS Statistics version 29 (IBM Corp., Armonk, N.Y., USA) and MedCalc, version 19.6.4 (MedCalc Software Ltd., Ostend, Belgium). The significance level was set at *P *< 0.05.

## Results

Nineteen patients (14 male, 5 female; ratio 2.8:1) were included in this study. The median follow-up time was 63 (IQR 34–104) months. The demographic data, clinical characteristics, and IMT at baseline are presented in Table 1. All patients were refractory to IMT and, in some cases, other anti-TNF-α agents. The mean age at the time of GLM initiation was 30.7 ± 11.0 years. All patients exhibited bilateral eye involvement. Granulomatous inflammation was reported in 14 eyes of 7 patients (36.8%; 5 patients with Vogt-Koyanagi-Harada disease, 2 patients with undifferentiated uveitis). Behçet’s disease was the most common diagnosis (57.9%), followed by Vogt-Koyanagi-Harada disease (26.3%) and psoriasis-associated panuveitis (5.3%). Two patients (10.5%) were diagnosed with undifferentiated panuveitis. At baseline, most patients had more than one IMT (73.7%), and alkylating agents combined with calcineurin inhibitors were the most frequent IMT (47.4%). During follow-up, none of the patients showed uveitis remission. The median follow-up time during GLM treatment was 20 (IQR 8–49) months, and the median number of GLM injections was 21 (IQR 8–15).

**Table 1:** Demographics and clinical characteristics of all patients at baseline.

	Total N = 19
Age (years), mean ± SD	30.7 ± 11
Sex, n (%)	
Male	14 (73.7)
Female	5 (26.3)
Duration of uveitis before receiving GLM (months), median (IQR)	55 (27–86)
Bilateral disease, n (%)	19 (100)
Granulomatous, n (%)	7 (36.8)
Diagnosis	
Behçet’s disease	11 (57.9)
VKH	5 (26.3)
Panuveitis associated with psoriasis	1 (5.3)
Undifferentiated panuveitis	2 (10.5)
Immunomodulatory drugs at baseline, n (%)	
CY + CsA	5
CHL + CsA	4
MMF alone	3
MTX + CY	2
MMF + CsA + CY	1
MTX + CsA	1
MMF + CsA	1
MTX alone	1
CY alone	1

*CHL* chlorambucil, *CsA* cyclosporin A, *CY* cyclophosphamide, *GLM* golimumab, *IQR* interquartile range, *MMF* mycophenolate mofetil, *MTX* methotrexate, *SD* standard deviation, *VKH* Vogt-Koyanagi-Harada disease.

### Uveitis relapse

**Figure 1:**
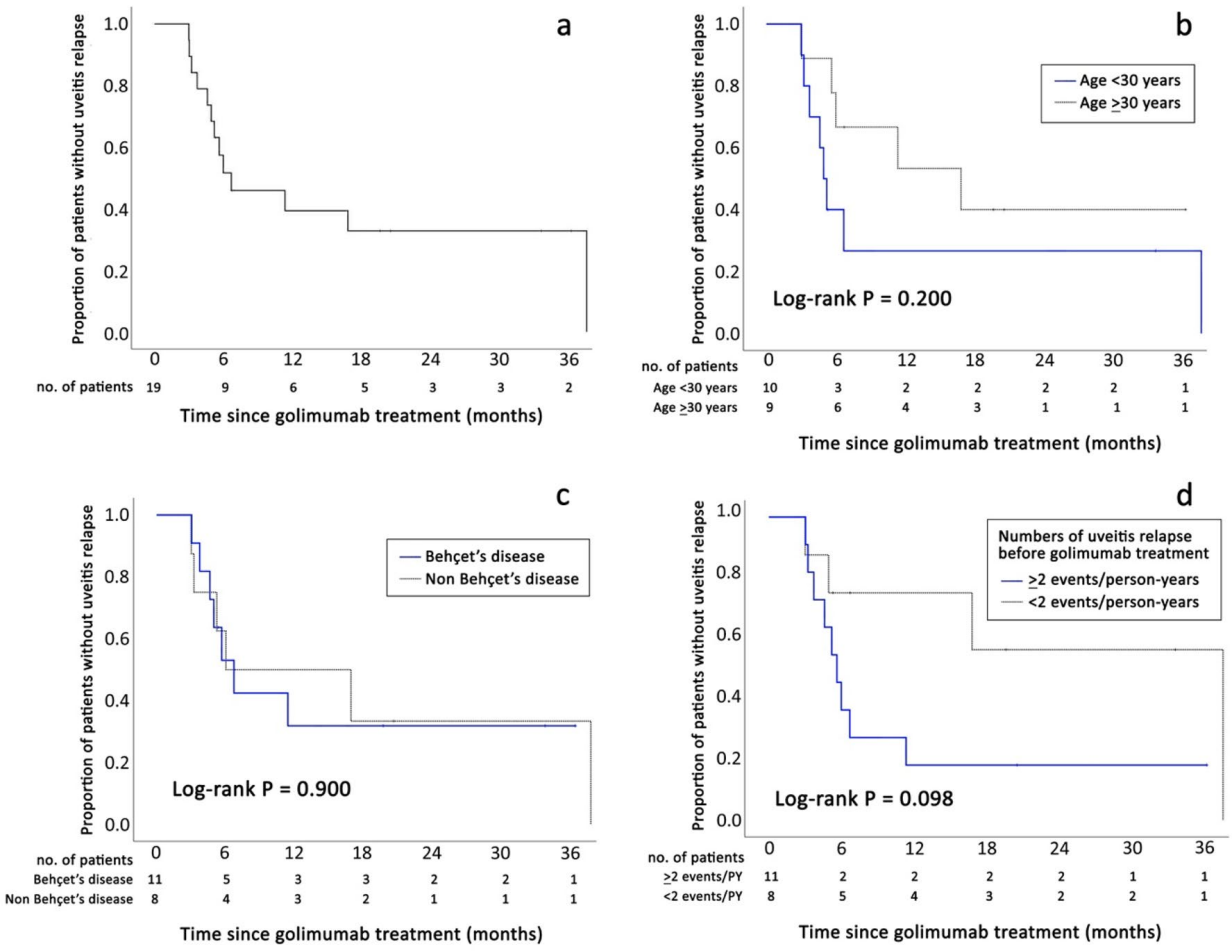
Kaplan–Meier curves showing the overall cumulative probability of the first uveitis relapse among 19 patients after golimumab treatment (**a**) and the association of the first uveitis relapse with age (**b**), Behçet’s disease (**c**), and number of uveitis relapses before golimumab treatment (**d**).

Before GLM treatment, 107 uveitis relapse events occurred over 61.82 PY, resulting in a relapse rate of 1.73 events/PY (95% confidence interval [95%CI] 1.42–2.09). After GLM treatment initiation, the number of relapse events decreased to 28 over 45.41 PY, resulting in a relapse rate of 0.62 events/PY (95%CI 0.41–0.89). Based on Poisson regression analysis, the uveitis relapse rate after GLM treatment was 0.33 times that before GLM treatment (95%CI 0.19–0.57, *P *< 0.001). After GLM treatment, 13 of the 19 patients experienced uveitis relapse. The probability of the first uveitis relapse following GLM treatment is illustrated in Fig. 1; the cumulative incidence rates of uveitis relapse were 11.0%, 54.1%, and 60.6% at 3, 6, and 12 months, respectively. Log-rank test analysis showed that patients aged < 30 years and those with ≥ 2 uveitis relapse events/PY before GLM treatment had higher rates of the first uveitis relapse following GLM treatment (*P *= 0.200 and *P *= 0.098, respectively). The rates of the first uveitis relapse did not differ between patients with Behçet’s disease and those with other uveitis types.

A Cox regression model was used to analyze the factors associated with the first uveitis relapse after GLM treatment. The results of univariate and multivariate logistic regression analyses are shown in Table 2. None of the variables in the univariate model, including age < 30 years, Behçet’s disease, and relapse ≥ 2 events/PY before GLM treatment were found to be factors significantly associated with the first uveitis relapse following GLM treatment. Age and number of relapse events before GLM treatment were subsequently analyzed using a multivariate model. Age < 30 years and relapse ≥ 2 events/PY before GLM treatment were associated with a higher rate of first uveitis relapse without reaching significance (adjusted odds ratio [aOR] 3.04, 95%CI 0.87–10.60, *P *= 0.082 and aOR 3.98, 95%CI 0.99–16.09, *P *= 0.053, respectively).

**Table 2:** Factors associated with the first uveitis relapse after golimumab treatment.

Characteristics	Univariate analysis		Multivariate analysis	
	Crude odds ratio (95%CI)	*P* value	Adjusted odds ratio (95%CI)	*P* value
Age < 30 years	2.12 (0.66, 6.81)	0.209	3.04 (0.87, 10.60)	0.082
Behçet’s disease	1.08 (0.34, 3.40)	0.900	–	
Relapse ≥ 2 events/PY before GLM treatment	2.91 (0.78, 10.89)	0.113	3.98 (0.99, 16.09)	0.053

*CI* confidence interval, *GLM* golimumab, *PY* person-years.

### Changes in visual acuity and corticosteroid and immunomodulatory agent dosages

**Figure 2:**
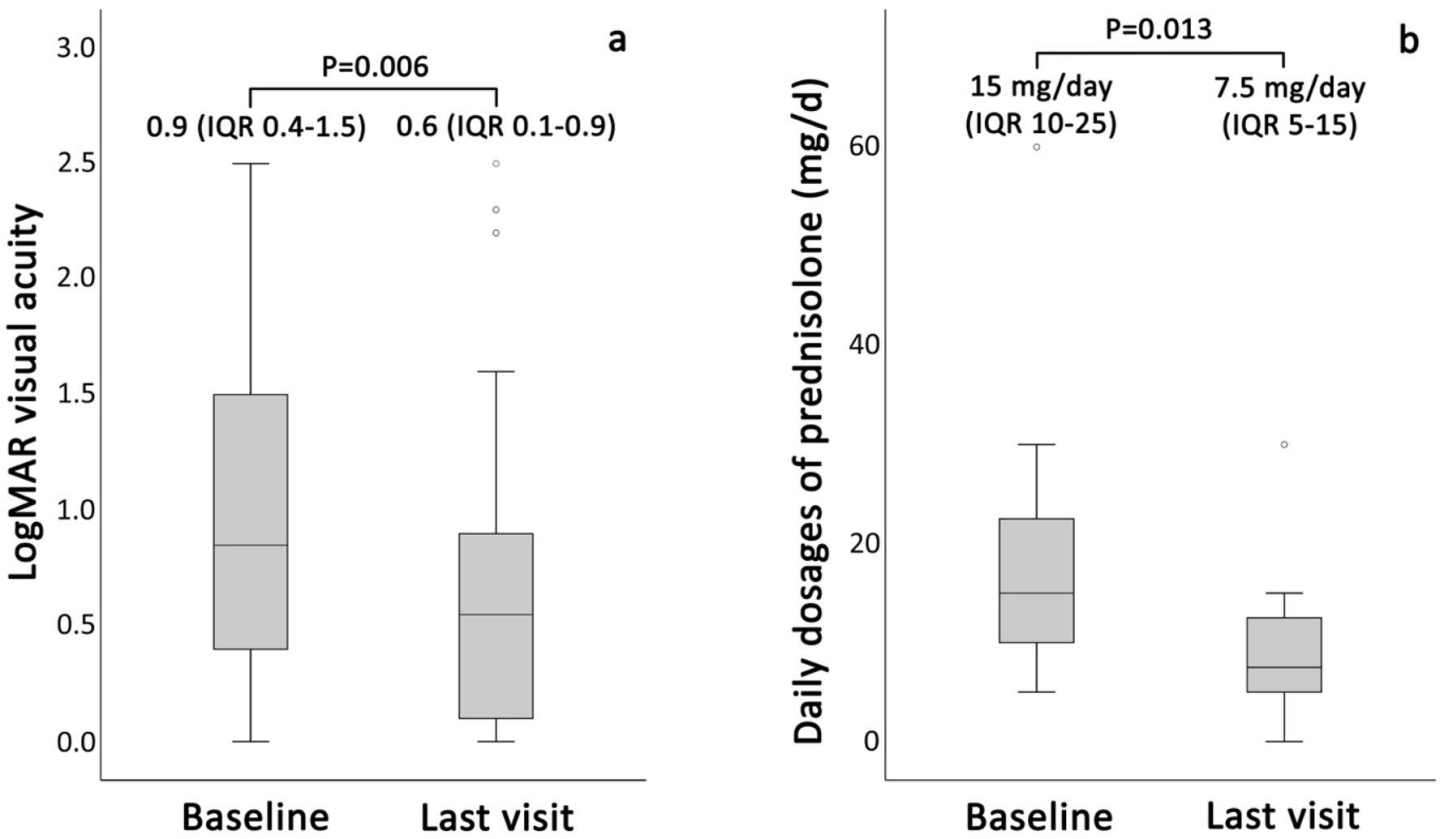
Median logMAR BCVA (**a**) and median dosage of oral prednisolone (mg/d) (**b**) at baseline and the last visit in patients with non-infectious refractory uveitis receiving golimumab. BCVA, best-corrected visual acuity; IQR, interquartile range; logMAR, logarithm of the minimum angle of resolution.

The logMAR BCVA values and dosages of systemic and topical prednisolone at specific time points are listed in Table 3. The median logMAR BCVA was 0.5 at both 3 and 6 months after GLM treatment, representing significant improvements from 0.9 at baseline (*P *= 0.021 and 0.027, respectively). At 12 months after GLM treatment, 7 patients (14 eyes) were lost to follow-up, and the median logMAR BCVA declined to the baseline value of 0.9 (*P *= 0.135). At the last visit, the median logMAR BCVA was 0.6, showing a significant improvement compared to the baseline value (*P *= 0.006; Fig. 2a).

**Table 3:** Visual acuity, prednisolone dosage, and topical prednisolone dosage at baseline, 3 months, 6 months, and 12 months follow-up.

	Baseline	3 months	6 months	12 months
Patients, n	19	19	19	12
Eyes, n	38	38	38	24
logMAR BCVA, median (IQR)	0.9 (0.4–1.5)	0.5 (0.2–1.0)	0.5 (0.2–1.1)	0.9 (0.3–1.6)
*P* value^a^		0.021	0.027	0.135
Prednisolone (mg/d), median (IQR)	15 (10–25)	12.5 (7.5–15)	10 (7.5–15)	6.3 (3.1–12.5)
*P* value^a^		0.091	0.038	0.013
Topical prednisolone (drops/d), median (IQR)	2 (0–6.5)	0 (0–2)	0 (0–2)	0 (0–1)
*P* value^a^		0.002	0.008	0.001

*BCVA* best-corrected visual acuity, *IQR* interquartile range, *logMAR* logarithm of the minimum angle of resolution.

^a^*P* value of Wilcoxon’s signed-rank test compared to baseline.

The median daily prednisolone dosages prescribed at baseline, 3 months, 6 months, and 12 months were 15.0, 12.5, 10.0, and 6.3 mg/d, respectively. The dosage was significantly reduced at 6- and 12-month follow-ups (*P *= 0.038 and 0.013, respectively). At the last visit, the median systemic prednisolone dosage was 7.5 mg/d which was half the baseline dosage (*P *= 0.013; Fig. 2b). The median dosage of prednisolone acetate 1% eye drops, which was 2 drops/d at baseline, decreased to 0 drops/d at 3, 6, and 12 months, and at the last visit (*P *= 0.001, 0.008, 0.001, and 0.280, respectively).

Regarding IMT use, 12 patients (63.2%) were able to reduce either the dosage or number of IMT agents following GLM treatment. Owing to differences in IMT agents and dosages, the treatment details of each patient are summarized in Supplemental Table 1.

### Adverse events

Among the 19 patients, three had to discontinue GLM due to adverse events. One patient with Behçet’s disease reported biopsy-confirmed GLM-induced psoriasiform dermatitis after 20 GLM doses. Another patient with undifferentiated granulomatous panuveitis developed disseminated varicella-zoster virus infection after 10 GLM doses. The third patient, who had Behçet’s disease, was newly diagnosed with primary vitreoretinal lymphoma after 8 GLM doses. This patient developed increased vitreous haze resistant to the current therapy with GLM, chlorambucil 5 mg/d, cyclosporine A 200 mg/d, and three doses of intravenous pulse methylprednisolone 1 g/d. Diagnostic pars plana vitrectomy was performed, and the pathological results revealed a diffuse large B-cell lymphoma.

## Discussion

This study evaluated 19 patients with refractory non-infectious panuveitis receiving monthly subcutaneous injections of 50 mg GLM. The rate of uveitis relapse was significantly lower after GLM treatment than before treatment. At the last visit, approximately three-fifths of patients were able to reduce the dosage or number of IMT agents, and the prednisolone dosage was halved. Moreover, significant VA improvements were observed at the last visit.

In this study under real-world conditions, the uveitis relapse rate after GLM treatment was 0.33 times that before treatment. This result signified a reduction from the pre-treatment rate, and this decrease is consistent with the findings of previous studies. Nevertheless, the rate is greater than those previously reported^6,9,11,12,15^. A PRISMA-compliant systematic review and meta-analysis of four studies reported a pooled hazard ratio of 0.05 (95%CI 0.00–1.38) for the relapse rate after GLM treatment^12^. However, only one of those four studies examined patients with refractory uveitis associated with Behçet’s disease (five patients), and analysing the uveitis relapse rates during the 12 months before and 12 months after GLM treatment, relapses occurred in their study in 11 and 1 patients, respectively (rate ratio = 0.09)^15^. Given the larger population and longer follow-up in our study, we believe that our uveitis relapse rates provide a more representative real-world picture of patients with refractory non-infectious panuveitis. Our findings suggest that GLM treatment may be an effective option for reducing uveitis relapse in this patient population.

Half of the patients experienced their first uveitis relapse within 6 months after initiating GLM treatment. We analysed factors potentially associated with the time of the first uveitis relapse after GLM treatment. After adjusting for confounders in multivariate analyses, no significant association was detected (*P *= 0.053), but patients with two or more uveitis relapse events/PY before GLM treatment had a higher relapse rate after receiving GLM. Thus, multiple uveitis relapses might be indicative of a severe disease course and increase the risk of subsequent relapses. However, a larger sample is required to confirm this. These findings highlight the importance of early GLM treatment in reducing the risk of further uveitis relapses. Early and effective treatment of patients with refractory non-infectious panuveitis is critical for improving patient outcomes and preventing further complications.

Moreover, patients aged < 30 years had a higher although not statistically significant relapse risk. This is consistent with the results of Celiker et al. who reported that older age reduces the relapse risk of Behçet’s uveitis^19^. Likewise, older age is associated with fewer relapses of neuro-Behçet’s syndrome^20^.

Behçet’s disease has been associated with a relapsing–remitting disease course which is considered more challenging, requiring more advanced therapies such as early administration of biologic agents. However, our study found no differences in uveitis relapse rates between patients with Behçet’s disease and those with other diseases, mainly Vogt-Koyanagi-Harada disease, possibly because all patients in our study had severe and chronic inflammation resistant to first- and second-line IMTs, as well as a history of multiple uveitis relapses. Therefore, all patients exhibited a similar uveitis relapse course, independent of their specific diagnosis.

A prednisolone dosage of ≤ 7.5 mg/d is generally considered physiological and can be prescribed with fewer systemic adverse effects, ensuring long-term safety. Reducing the prednisolone dosage to ≤ 10 mg/d while preventing uveitis relapses is primarily considered a successful corticosteroid-sparing therapy^17^. We observed a significantly reduced median dosage of oral prednisolone in patients receiving GLM, resulting in a decreased dosage of < 10 mg/d at the last visit. This is consistent with results from other studies, which also demonstrated that GLM reduces the dosage of oral corticosteroids required to manage NIU^6,7,11,15^.

Interestingly, four out of the seven patients without decreased IMT dosages or numbers after GLM treatment had a history of infliximab treatment. Jin et al. investigated the efficacy of GLM in refractory NIU, and their subgroup analyses revealed that 7 out of 30 patients with a history of anti-TNF-⍺ inhibitor treatment were related to decreased GLM efficacy, although without reaching statistical significance. Antibodies directed against anti-TNF-⍺ inhibitors might affect the GLM efficacy in such patients^16^.

In the present study, patients with refractory uveitis had poor VA (median logMAR 0.9; Snellen equivalent 20/160) before GLM treatment. We hypothesize that as most patients experienced multiple uveitis relapses, intraocular inflammation and damage to the optic nerve and retina had repeatedly occurred over a considerable time prior to GLM treatment. However, we observed a significant VA improvement at 3 and 6 months after GLM treatment. At 12 months, the median VA had deteriorated to the baseline level. Our analysis showed that most patients with better VA had left the study. Three were lost to follow-up, and four had switched to adalimumab, which was not due to a lack of efficacy but rather to lower costs. At the last visit, a three-line VA improvement compared to baseline was achieved by GLM treatment which is similar to the results of other studies on non-infectious panuveitis^14–16^.

Based on the SUN criteria, none of the patients in this study achieved uveitis remission, as all had to continue uveitis treatment^17^. Although systemic corticosteroids could be tapered off in two patients, all patients still needed at least one IMT agent at the last follow-up visit. If we consider controlled uveitis as grade 0 anterior chamber cells, the controlled uveitis rate at the last visit was 42.1%.

Regarding adverse events, none of the patients had elevated liver enzyme levels requiring GLM discontinuation during follow-up. The safety profile of GLM is reported to be comparable to that of other anti-TNF-⍺ agents^5^. This drug class is associated with an increased risk of opportunistic infections as seen in one patient of this study who developed disseminated varicella-zoster virus infection. Another concern was lymphoma, as one patient developed primary vitreoretinal lymphoma. In our literature search, we found a similar case report, in which primary vitreoretinal lymphoma developed in a young woman with rheumatoid arthritis and uveitis who had received longstanding treatment with methotrexate and TNF-⍺ inhibitors (etanercept, infliximab, GLM)^21^. The development of this secondary malignancy in one of our patients is a rare and serious adverse event that may warrant further investigation into the possible association with GLM treatment.

One patient with Behçet’s disease developed GLM-induced psoriasiform dermatitis, which is less frequently reported than for infliximab or adalimumab treatment^22–25^. Patients with a history of psoriasis should be closely monitored during GLM treatment. Interestingly, the patients with psoriasis-associated panuveitis included in our study did not experience worsening dermatitis under GLM treatment.

This study has some limitations, including its retrospective design, lack of a control group, and non-standardised follow-up duration. The limited number of enrolled patients may have introduced a selection bias, influencing the outcomes. However, our sample size was relatively large, and the follow-up period was longer than those of other studies^14–16^. Overall, we believe that the findings of this study provide important support for the efficacy of GLM as an alternative treatment option for refractory non-infectious panuveitis.

In conclusion, GLM administration effectively reduced the inflammatory relapse rate and the use of systemic corticosteroids and conventional IMTs in patients with non-infectious panuveitis. These findings suggest that GLM is an assuring treatment option for patients with refractory non-infectious panuveitis, particularly in those with Behçet’s or Vogt-Koyanagi-Harada disease which comprised the majority of cases in the present study.

### Supplementary Information

Supplementary Table 1.

## Data Availability

The datasets used and/ or analyzed during the current study are available from the corresponding author on reasonable request.
