# Supplementary material for: Efficacy of golimumab in patients with refractory non-infectious panuveitis
Source: Sci Rep. 2024 Jan 25;14:2179. doi: 10.1038/s41598-024-52526-1 (PMC10811229; doi:10.1038/s41598-024-52526-1)
Supplement: Supplementary file 1 — Supplementary Table 1. [file 41598_2024_52526_MOESM1_ESM.docx]

**Supplemental Table 1.** Summary details of all 19 patients.

| No. | Sex | Age (year) | Diagnosis | Prednisolone dosage (mg) at baseline/last visit | | All IMT/biologics received before GLM | IMT/biologics at baseline | IMT/biologics at last visit | Total GLM injections |
| --- | --- | --- | --- | --- | --- | --- | --- | --- | --- |
| 1 | M | 28 | Behçet’s disease | | 20/2.5 | MTX, MMF, CsA, TAC, CY, CHL, IFX | MMF 1.5 g/d | GLM, AZA 100 mg/d | 57 |
| 2 | M | 38 | Behçet’s disease | | 15/15 | MMF, AZA, CsA, IFX | MMF 1.5 g/d | GLM, MMF 1.5 g/d, CY 50 mg/d | 15 |
| 3 | M | 34 | Behçet’s disease | | 30/10 | MTX, AZA, CsA, IVCY, CHL | CY 100 mg/d, CsA 100 mg/d | GLM, MTX 25 mg/week | 54 |
| 4 | M | 32 | Behçet’s disease | | 10/10 | MTX, CY, CHL | CY 100 mg/d, MTX 10 mg/week | GLM, MMF 2 g/d, CsA 50 mg/d | 27 |
| 5 | M | 24 | Behçet’s disease | | 10/5 | MTX, AZA, CY | CY 100 mg/d, MTX 7.5 mg/week | GLM, MTX 10 mg/week | 51 |
| 6 | M | 22 | Behçet’s disease | | 15/30 | CsA, CY, IFX | CY 100 mg/d CsA 150 mg/d | GLM, CsA 200 mg/d, CHL 5 mg/d | 8 |
| 7 | M | 24 | Behçet’s disease | | 5/0 | MMF, CsA | MMF 3 g/d | GLM, MMF 2 g/d | 27 |
| 8 | M | 40 | Behçet’s disease | | 60/5 | CsA, CHL | CHL 4 mg/d, CsA 200 mg/d | GLM, CHL 6 mg/d | 33 |
| 9 | M | 41 | Behçet’s disease | | 60/5 | MTX, AZA, CsA, CY, CHL | CHL 4 mg/d, CsA 25 mg/d | GLM, CHL 4 mg/d | 20 |
| 10 | M | 27 | Behçet’s disease | | 25/15 | MTX, AZA, CsA, CY | MTX 25 mg/week, CsA 300 mg/d | GLM, MTX 25 mg/week, CsA 200 mg/d | 6 |
| 11 | M | 22 | Behçet’s disease | | 20/7.5 | CY | CY 150 mg/d | GLM, CY 125 mg/d | 8 |
| 12 | F | 14 | VKH | | 20/30 | MTX, CsA, IVCY | CsA 200 mg/d, IVCY 1500 mg/month | GLM, CHL 8 mg/d | 51 |
| 13 | M | 45 | VKH | | 10/5 | CsA, CY, CHL, IVCY | CsA 200 mg/d, CHL 8 mg/d | GLM, CHL 4 mg/d | 31 |
| 14 | F | 23 | VKH | | 12.5/15 | CsA, CY, CHL | CHL 5 mg/d, CsA 50 mg/d | GLM, CHL 7 mg/d, CsA 150 mg/d | 8 |
| 15 | M | 55 | VKH | | 7.5/0 | MTX, MMF, AZA, CsA, CY, CHL | CsA 100 mg/d, CY 100 mg/d | GLM, CsA 50 mg/d, CY 50 mg/d | 21 |
| 16 | F | 45 | VKH | | 10/7.5 | MTX, MMF, CsA, TAC, CY | MMF 2 g/d, CsA 100 mg/d, CY 100 mg/d | GLM, CsA 50 mg/d, CY 100 mg/d | 12 |
| 17 | F | 17 | Panuveitis associated with psoriasis | | 10/5 | MTX, AZA, IFX | MTX 10 mg/week | GLM, MTX 20 mg/week | 66 |
| 18 | F | 20 | Undifferentiated panuveitis | | 25/10 | MMF, CsA, CHL | MMF 1 g/d, CsA 100 mg/d | GLM alone | 10 |
| 19 | M | 32 | Undifferentiated panuveitis | | 10/7.5 | MTX, AZA, CsA | CsA 150 mg/d, CY 100 mg/d | GLM, CsA 150 mg/d, CY 100 mg/d | 7 |

*AZA* azathioprine, *CHL* chlorambucil, *CsA* cyclosporine A, *CY* cyclophosphamide, *F* female, *GLM* golimumab, *IFX* infliximab, *IMT* immunomodulatory drug, *IVCY* intravenous cyclophosphamide, *M* male, *MMF* mycophenolate mofetil, *MTX* methotrexate, *TAC* tacrolimus, *VKH* Vogt-Koyanagi-Harada disease.
